# Quantitative assessment of the generalizability of a brain tumor Raman spectroscopy machine learning model to various tumor types including astrocytoma and oligodendroglioma

**DOI:** 10.1117/1.JBO.30.1.010501

**Published:** 2025-01-24

**Authors:** Frédéric Leblond, Frédérick Dallaire, Katherine Ember, Alice Le Moël, Victor Blanquez-Yeste, Hugo Tavera, Guillaume Sheehy, Trang Tran, Marie-Christine Guiot, Alexander G. Weil, Roy Dudley, Costas Hadjipanayis, Kevin Petrecca

**Affiliations:** a Polytechnique Montréal, Department of Engineering Physics, Montreal, Quebec, Canada; b Centre de recherche du Centre hospitalier de l’Université de Montréal, Montreal, Quebec, Canada; c Institut du cancer de Montréal, Montreal, Quebec, Canada; d Montreal Neurological Institute-Hospital, Division of Neuropathology, Department of Pathology, Montreal, Quebec, Canada; e Centre hospitalier universitaire Sainte-Justine, Department of Neurosurgery, Montréal, Quebec, Canada; f McGill University Health Center, Department of Neurosurgery, Montréal, Quebec, Canada; g University of Pittsburgh Medical Center, Pittsburgh, Pennsylvania, United States; h McGill University, Montreal Neurological Institute-Hospital, Montreal, Quebec, Canada

**Keywords:** Raman spectroscopy, fluorescence, machine learning, biomedical optics, tissue optics, biochemistry, brain cancer, neurosurgery, gliomas, glioblastoma

## Abstract

**Significance:**

Maximal safe resection of brain tumors can be performed by neurosurgeons through the use of accurate and practical guidance tools that provide real-time information during surgery. Current established adjuvant intraoperative technologies include neuronavigation guidance, intraoperative imaging (MRI and ultrasound), and 5-ALA for fluorescence-guided surgery.

**Aim:**

We have developed intraoperative Raman spectroscopy as a real-time decision support system for neurosurgical guidance in brain tumors. Using a machine learning model, trained on data from a multicenter clinical study involving 67 patients, the device achieved diagnostic accuracies of 91% for glioblastoma, 97% for brain metastases, and 96% for meningiomas. Here, the aim is to assess the generalizability of a predictive model trained with data from this study to other types of brain tumors.

**Approach:**

A method was developed to assess the generalizability of the model, quantifying performance for tumors including astrocytoma, oligodendroglioma and ependymoma, pediatric glioblastoma, and classification of glioblastoma data acquired in the presence of 5-ALA induced fluorescence. Statistical analyses were conducted to assess the impact of vibrational bands beyond contributors identified in our previous research.

**Results:**

A machine learning brain tumor detection model showed a positive predictive value (PPV) of 70% for astrocytoma, 74% for oligodendroglioma, and 100% for ependymoma. Furthermore, the PPV was 100% in classifying spectra from a pediatric glioblastoma and 90% for detecting adult glioblastoma labeled with 5-ALA-induced fluorescence. Univariate statistical analyses applied to individual vibrational bands demonstrated that the inclusion of Raman biomarkers unexploited to date had the potential to improve detectability, setting the stage for future advances.

**Conclusions:**

Developing predictive models relying on the inelastic scattering contrast from a wider pool of Raman bands may improve detection accuracy for astrocytoma and oligodendroglioma. To do so, larger tumor datasets and a higher Raman photon signal-to-noise ratio may be required.

Completeness of resection is a critical factor in glioma patients’ outcomes as more extensive removal of tumor tissue is associated with improved survival, decreased risk of recurrence, and improved treatment response.^1^^,^^2^ The most aggressive brain tumor is glioblastoma, representing 50% of all gliomas, whereas oligodendroglioma and astrocytoma account for 30%.^3^ Surgical success in glioma surgery depends on the ability to maximize the volume of cancer tissue removed while minimizing damage to perilesional normal tissue. Several technologies are used during glioma surgery, including neuronavigational systems providing 3D imaging-based guidance to help surgeons navigate through the brain. 5-ALA fluorescence-guided surgery (FGS) is also used as an aid in tumor visualization for glioblastomas. However, its utility for World Health Organization (WHO)-grade II astrocytoma and oligodendroglioma, i.e., low-grade gliomas, is limited.^4^

We developed a technique using Raman spectroscopy that allows live tissue characterization in real time during neurosurgery.^5^^,^^6^ The system was initially developed as a laboratory instrument but underwent several evolutionary steps over a decade, culminating with the Sentry system manufactured by Reveal Surgical [Fig. 1(a)]. The system is composed of a light illumination system and a spectroscopic sensing unit [Fig. 1(b)] connected to a sterilizable hand-held probe [Fig. 1(c)]. It was developed to acquire the spectroscopic tissue signature of a single point covering a circular surface area with a diameter of 500  μm. The signal is acquired within 5 s following excitation using a near-infrared 785-nm laser. Spectroscopic detection of reemitted light is achieved in the range 800 to 950 nm with a spectral resolution of ∼0.5  nm. The cumulative light dose used per measurement is less than the maximum permissible exposure for skin as set by laser safety guidelines from the American National Standards Institute.^7^

**Fig. 1:**
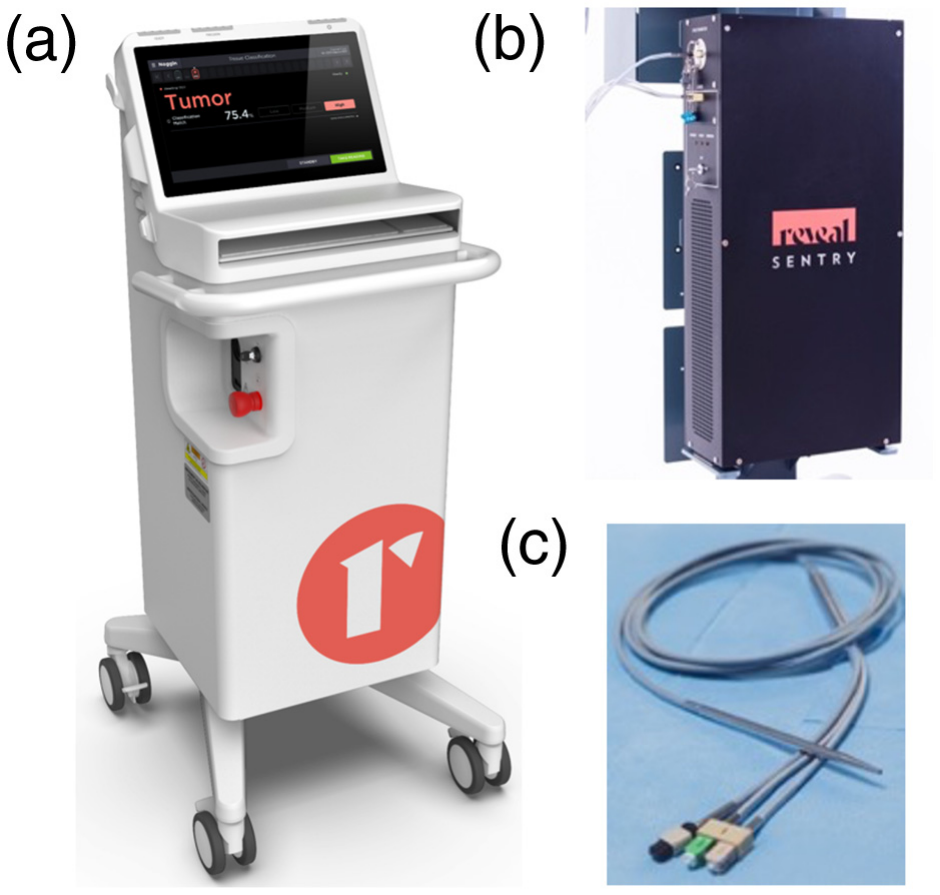
(a) Intraoperative Raman spectroscopy system used in the scope of the study that was designed for *in situ* tissue sensing using a hand-held fiber-optics probe, (b) illumination and detection sub-system, (c) fiber-optics probe.

The detected signal is composed of a large amplitude background overlayed with lower intensity contributions from inelastically scattered diffused Raman photons.^8^ The overall magnitude of the detected Raman signal is impacted by several factors. These include intrinsic tissue optical properties associated with absorption from endogenous brain chromophores (e.g., hemoglobin) and elastic scattering from microscopic tissue structures such as cell membrane and organelle lipid bilayers.^9^ The signal-to-noise ratio (SNR) associated with Raman photons is also impacted by background photonic counts from endogenous tissue fluorescence, which can be orders of magnitude larger when compared with the Raman signal.^10^ As optical properties of tissue vary between tissue types, different Raman spectroscopy applications will lead to different light detection challenges. As such, optimized imaging parameters for light exposure, integration time, and number of repeat measurements will be required to acquire sufficient Raman SNR levels for a given detection task. For example, an application requiring discrimination between fatty tissue and protein-rich structures may only require the detection of the high-intensity protein/lipid band at a wavenumber shift of $1441\;{\mathrm{cm}}^{-1}$ without the need to optimize Raman SNR in other bands that have intrinsically lower levels of inelastic scattering.^11^ However, the detection of more subtle disease signatures may require sensing of lower amplitude bands, thus providing more detailed information. More subtle signatures could include those associated with nucleic acids (DNA and RNA) or motifs associated with the secondary structure of proteins.^12^

In Raman spectroscopy, the signal of interest is isolated from a largely featureless background using curve-fitting algorithms, exploiting the fact that inelastic scattering is associated with sharp peaks sourced by specific molecular vibrational bonds.^13^^,^^14^ Following standard calibration and normalization procedures, the result is a spectrum providing the relative intensity of all inelastic scattering bands (visualized as peak height on the y-axis) and peak position along an x-axis associated with wavenumber shifts from 785 nm, historically reported in units of inverse centimeters (${\mathrm{cm}}^{-1}$). The spectra can then be normalized, a common approach being standard normal variate normalization. The resulting signal then provides a vibrational spectroscopic fingerprint of the tissue in which the height of each peak, rather than being an absolute measure of the molecular bonds, represents the relative concentration of biomolecules compared with the overall Raman-predicted molecular content of the tissue.^15^

The Sentry system was used to conduct a multicenter study involving 67 adult brain tumor patients with glioblastoma, meningioma, or brain metastases. A total of 1329 spectra were acquired with tissue samples for each.^16^ Machine learning models were developed to detect bulk tumors that were either glioblastoma, meningioma, or metastatic disease. These models were trained on a data subset consisting of 80% of the whole dataset and were tested on an independent holdout set consisting of the remaining ensemble of data. The sensitivity/specificity of detection of these models, based on predictions from the holdout data subsets, was 91%/91% for glioblastoma, 97%/98% for meningioma, and 96%/95% for metastases. These results generated from a commercial system were consistent with previous studies using a laboratory system.^17^

A key finding from the multicenter study performed with Sentry was that the brain tumor detection machine learning models exploited only a subset of all available information in the spectra. Only four spectral features were required to generate a clear tissue-discriminatory biomolecular signature. The biomarkers and associated spectral features were protein (phenylalanine) at $1004\;{\mathrm{cm}}^{-1}$, protein (tryptophan) at $1340\;{\mathrm{cm}}^{-1}$, lipids at $1299\;{\mathrm{cm}}^{-1}$, and the lipid and protein peak at $1441\;{\mathrm{cm}}^{-1}$.^15^ In the multicenter study, the bulk tumor was associated with an increase in the height of peaks associated with protein and a reduction in peaks associated with lipids. This is consistent with the biochemistry of tumors, in which healthy lipid-rich brain tissues give way to fibrotic protein-rich tumor tissue.

The bio-informational content from brain-derived Raman spectra is rich. It includes more than 40 peaks associated with vibrational bonds that can be used as surrogates for the presence of different families of biomolecules.^16^ For example, a subset of peaks can be used to identify protein or lipid molecular bonds independent of the specific type of protein or lipid. Other peaks can provide more specific information about amino acids either in their free form or within proteins, e.g., the aromatic amino acids phenylalanine, tryptophan, and tyrosine. The idea that this wide variety of biomolecular information could be reliably accessed live during surgery is enticing. It could open the door not only to the identification of gross features associated with bulk tumors but could also be exploited to inform on subtle phenomena allowing tumor stratification in terms of their primary origin and grade.

Here, we present results supporting the fact that the biomolecular signature captured in our previous work can be used to detect multiple types of brain tumors. Raman spectroscopy data were acquired during surgery for adults with astrocytoma, oligodendroglioma and ependymoma, a pediatric glioblastoma, and adult patients undergoing 5-ALA fluorescence-guided glioblastoma surgery (Table S1 in the Supplementary Material). The latter is particularly important if intraoperative Raman spectroscopy is to become a new standard-of-care during glioblastoma surgery, either as a stand-alone detection system or with 5-ALA fluorescence-guided surgery.^12^ Thirty-one spectra were acquired from three patients with astrocytoma (WHO grade III), 30 spectra from three patients with oligodendroglioma (two patients WHO Grade II, one patient WHO Grade III), and six spectra from one patient with ependymoma (WHO Grade II). Furthermore, 33 spectra were acquired from nine glioblastoma patients undergoing 5-ALA FGS. Thirteen spectra were also acquired from two pediatric patients: four tumor spectra with glioblastoma and nine spectra from a focal cortical dysplasia (FCD) patient. The nine spectra from the FCD patient were acquired in areas of the non-tumoral brain. Pediatric measurements were made at the McGill University Health Center Children’s Hospital. The spectra from oligodendroglioma, astrocytoma, and ependymoma were acquired at the Montreal Neurological Institute-Hospital, and the adult glioblastoma measurements with 5-ALA were completed at the Mount Sinai Hospital in New York City.

A brain tumor detection machine learning model was developed using data acquired from our previous multicenter study that relied on spectral features extracted from the bands with center wavenumber values at 1004, 1299, 1340, and $1441\;{\mathrm{cm}}^{-1}$.^16^ Specifically, the training set consisted of 101 tumor spectra and 148 non-tumoral brain spectra from 13 metastases patients, and 366 tumor spectra and 185 non-tumoral brain spectra were acquired from 26 patients with glioblastoma (Table S2 in the Supplementary Material: first and second lines for glioblastoma). Unbalanced classes in each model are accounted for with a class weight parameter adjusted to reflect the ratio between non-tumoral and tumoral brain samples.^18^ Prior to model training, all spectra were checked for quality. Low Raman SNR spectra (spectra dominated by stochastic noise) and spectra plagued with spectral artifacts unrelated to the tissue signature, such as ambient light artifacts, were removed from the dataset.^19^ This led to a training set composed of 370 tumor measurements and 232 non-tumoral brain measurements [Fig. 2(a)]. The quantitative differences between non-tumoral brain and tumor spectra were pronounced and unambiguous for the Raman intensity bands exploited by machine learning. When taken by themselves as individual biomarkers, the intensity of each of the bands at 1004, 1299, 1340, and $1441\;{\mathrm{cm}}^{-1}$ led, without machine learning, to accuracy/sensitivity of 80%/80%, 81%/80%, 80%/79%, and 87%/86%, respectively (Table S2 in the Supplementary Material).

**Fig. 2:**
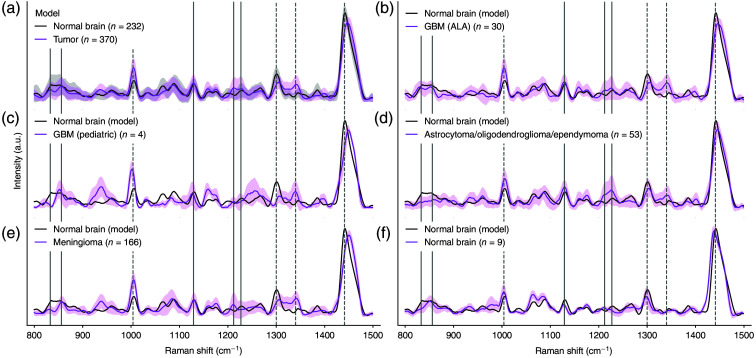
(a) Raman spectra used to train and validate a tumor detection machine learning model discriminating between non-tumoral brain and tumor tissue. Spectra used to evaluate the generalizability of the model: (b) glioblastoma measurements made during FGS, (c) glioblastoma measurements in a pediatric patient, (d) adult astrocytoma, oligodendroglioma, and ependymoma. The third row (e) shows the spectra associated with meningioma measurements and (f) the non-tumoral brain measurements acquired in an epilepsy patient. The spectrum associated with the average of all non-tumoral brain measurements used to train the model is shown as magenta dotted lines. All spectral features used in the scope of the cancer detection model are identified with vertical dotted lines. Plain vertical lines highlight bands for which univariate statistical analyses were performed to compare tumor measurements with non-tumoral brain (Fig. 3). Shaded areas represent the variance computed for each Raman intensity across all measurements.

Machine learning was required to automatize the simultaneous use of multiple features to exploit their synergistic value. The training and validation methods have been described elsewhere.^16^ Briefly, they consist of dimensionally reducing the dataset based on an L1-normalization approach relying on support vector machines (SVMs). The resulting features, associated with the intensity from specific vibrational bonds, were then used to train SVM tumor detection models using five-fold cross-validation based on a leave-one-patient-out approach. Receiver operating characteristic (ROC) analyses were performed leading to the selection of one model that optimized sensitivity and specificity. The results for the training phase led to a ROC curve area under the curve of 0.96 with different values of sensitivity and specificity obtained by varying the curve parameter λ. The threshold value for λ was selected as corresponding to the point closest to the upper left corner of the ROC curve (y-axis: sensitivity, x-axis: 1—specificity). This led to a training sensitivity of 90% and a specificity of 91%.

In a previous work, four tumor models were developed: glioblastoma, metastases, meningioma, and one including all three tumor types. Here, we present a model based on combined data from glioblastoma and metastases. The meningioma dataset was voluntarily kept aside for model testing to quantitatively assess the generalizability of new data (Table S1 in the Supplementary Material: last line). To further assess its generalizability, the brain tumor model was directly applied to all spectra from astrocytoma, oligodendroglioma, ependymoma, pediatric glioblastoma, 5-ALA labeled adults, and non-tumoral brain measurements from the FCD patient (Fig. 2). For each spectrum, this resulted in a prediction of *tumor* or *non-tumor*.

Prior to applying the tumor model on the independent dataset, all spectra were checked for quality to ensure that no low Raman SNR spectra were kept, resulting in 85% of spectra being retained (numbers in parentheses in Table S1 in the Supplementary Material).^19^ All 166 meningioma spectra were classified as tumors, resulting in a positive predictive value (PPV) of 100%. The PPV for astrocytoma, oligodendroglioma, and ependymoma was 70%, 74%, and 100%, respectively. All four tumor spectra acquired from the pediatric glioblastoma patient were predicted to be cancer (PPV = 100%), and of the 30 spectra from the 5-ALA labeled glioblastomas, 27 were predicted as tumor (PPV = 90%). Finally, all spectra from the FCD patient were predicted as non-tumoral brain, resulting in a negative predictive value of 100%. These results show that the model generalized well to most adult brain tumor types, in 5-ALA fluorescence-guided surgery and conventional surgery. The PPV of the model for astrocytoma and oligodendroglioma is <75%. The underperformance of the model can likely be traced back to the need for the inclusion of more biomolecular features during the model training phase to fully capture the key spectral differences between these tumors and the non-tumoral brain. No measurements from these types were used to train the cancer detection model.

To preliminarily assess whether Raman peaks beyond the four used to develop the tumor detection model could improve the detection of astrocytoma and oligodendroglioma from the non-tumoral brain, univariate analyses (Kruskal–Wallis test) were performed on a larger pool of spectral features. These analyses allowed pairwise comparisons quantifying statistical significance using 11 Raman bands for which a clear biochemical interpretation could be provided (Fig. 3).^16^ The spectral bands considered were at wavenumber shifts 833, 856, 1129, 1159, 1175, 1212, and $1227\;{\mathrm{cm}}^{-1}$. Analyses were also performed for the four bands associated with the tumor model, i.e., at 1004, 1299, 1340, and $1441\;{\mathrm{cm}}^{-1}$.

**Fig. 3:**
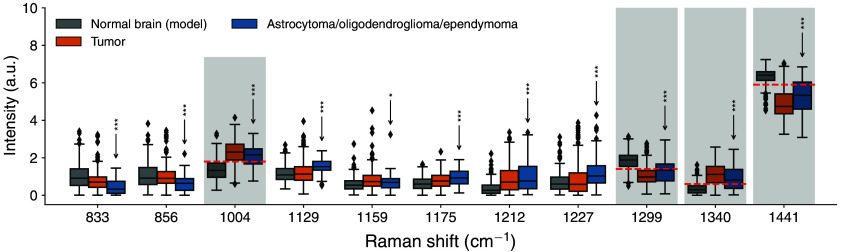
Box and whisker plots associated with a subset of all Raman bands that were identified on brain spectra and for which a biomolecular interpretation was provided. The bands that were retained by the machine learning brain tumor detection model are represented by shadowed areas: 1004, 1299, 1340, and $1441\;{\mathrm{cm}}^{-1}$. The dotted red lines are the threshold values used to compute the accuracy, sensitivity, and specificity of tumor detection for individual bands (Table S1 in the Supplementary Material). The other bands are associated with Raman shifts at 833, 856, 1129, 1159, 1175, 1212, and $1227\;{\mathrm{cm}}^{-1}$. Univariate statistical analyses were performed allowing pairwise comparisons between spectra data acquired in non-tumoral brain and data acquired in tumor tissue: *p<0.05, **p<0.01, and ***p<0.001.

The statistical analyses revealed that there are statistically significant differences for all considered bands between the non-tumoral brain and astrocytoma, oligodendroglioma, and ependymoma pooled together. Up- or down-regulation of the $833/856\;{\mathrm{cm}}^{-1}$ bands was associated with the amino acid tyrosine, whereas the bands in the 1129 to $1175\;{\mathrm{cm}}^{-1}$ range were associated with the C-C bonds of lipids. The band at $1129\;{\mathrm{cm}}^{-1}$ has been related to nucleic acid phosphates. The $1212/1227\;{\mathrm{cm}}^{-1}$ bands have been linked with the amide content of proteins, as well as unsaturated lipids.^16^ In several instances, the boxes in the whisker plots were clearly separated, with limited overlap between the interquartile range associated with the non-tumoral brain when compared with tumor tissue.

A trait that is common across all newly considered bands is that they are associated with intrinsically lower inelastic scattering signals when compared with bands used by the tumor detection model. Those lower signal bands were associated with lower photon counts and generally also with a lower ratio of Raman scattering to the overall fluorescence background. Thus, they typically had a higher level of stochastic noise, i.e., a reduced Raman SNR. This points to an intrinsic limitation of the spectroscopic dataset as it relates to the detected levels of Raman photon SNR within the seven bands for which there were statistically significant differences between astrocytoma—oligodendroglioma and ependymoma—and non-tumoral brain. In fact, the level of inter-measurement variance associated with those bands, when compared with the SNR associated with the four bands considered part of the brain tumor model, is overall more pronounced. We hypothesize that minimizing the stochastic noise within detected spectra may allow the development of more accurate predictive models.

Our work shows the broad generalizability of the Sentry system and machine learning models to multiple types of brain cancers, including meningioma, oligodendroglioma, ependymoma, astrocytoma, and pediatric glioblastoma. It is also agnostic to the presence of 5-ALA-induced fluorescence. Increasing Raman SNR in future work is a realistic endeavor, which may greatly increase the available informational content when developing predictive models. A limitation of the current dataset was that all spectra were acquired with a fixed laser power, a fixed integration time, and a fixed number of repeat measurements per point. This led to a large variability in absolute detected photonic counts per measurement, leading to an unequal distribution of stochastic noise levels across the dataset, especially for those intrinsically lower intensity bands. However, the more recent version of the Raman system integrates automated integration time adjustments, ensuring consistency of overall photonic counts detected per measurement by maximizing usage of charged-coupled device sensor dynamical range.^10^

## Supplementary Material



## Data Availability

The data and material information that support the findings of this study are available from the corresponding author upon reasonable request.
