# Supplementary material for: Quantitative assessment of the generalizability of a brain tumor Raman spectroscopy machine learning model to various tumor types including astrocytoma and oligodendroglioma
Source: J Biomed Opt. 2025 Jan 24;30(1):010501. doi: 10.1117/1.JBO.30.1.010501 (PMC11758428; doi:10.1117/1.JBO.30.1.010501)
Supplement: Supplementary file 1 [file JBO_030_010501_SD001.pdf]

Table S1

Peak	AUC	Accuracy	Sensitivity	Specificity	PPV
1004	0.85	80%	80%	80%	68%
1299	0.81	81%	80%	83%	58%
1340	0.87	80%	79%	83%	60%
1441	0.87	87%	86%	88%	68%

Table S2

Cancer type	Number of patients		Normal brain		Tumor	
Metastasis	13	(13)	148	(108)	101	(88)
Glioblastoma (adult)	26	(24)	185	(124)	366	(282)
Glioblastoma (pediatric)	2	(2)	9	(9)	4	(4)
Glioblastoma (ALA)	9	(9)	—	—	33	(30)
Oligodendroglioma	3	(3)	—	—	30	(27)
Astrocytoma	3	(3)	—	—	31	(20)
Ependymoma	1	(1)	—	—	6	(6)
Meningioma	8	(8)	—	—	180	(166)
